# Comments on: Differential IgG4-Producing Plasma Cell Infiltration in Non- and Post-Transplant Plasma Cell Hepatitis

**DOI:** 10.3389/ti.2022.10590

**Published:** 2022-08-05

**Authors:** Isabel Aguilera, Jose Manuel Sousa

**Affiliations:** ^1^ Immunology, Instituto de Biomedicina de Sevilla (IBIS), Hospital Universitario Virgen del Rocío, Seville, Spain; ^2^ Digestive Diseases Service, Hospital Universitario Virgen del Rocío, Seville, Spain

**Keywords:** IgG4, PC-rich R, GSTT1, liver transplant, plasma cells

We read with interest the article by Horwich et al. about IgG4-producing plasma cells. The aim of the study was to use IgG4-positivity as a differential biomarker for distinct clinical presentations of plasma cell hepatitis before and after liver transplantation. They found a high degree of IgG4-PC infiltration more frequently associated with plasma cell rejection (PCR) than other types of AIH and concluded that IgG4-positivity might serve as a valuable diagnostic tool in the post-LT setting.

It is very gratifying to see a confirmation of our previous report regarding the presence of IgG4 PCs in plasma cell-rich rejection (PC-rich R) biopsies. Our group identified the cellular profile associated with PC-rich R, and quantified the number of cells per mm^2^ of tissue by using a Computer-Assisted System Technology (newCAST™). The relative proportion of the main cell types was assessed. The results showed an important representation of IgG4^+^ PCs with a mean value of 5.9% (0.5%–19.8%) of the total number of immune cells in the inflammatory infiltrates found in portal areas (1).

A search in the scientific literature is complicated since *de novo* autoimmune hepatitis, first described in 1998 (2), has received many different names throughout these years until, in a recent update, the Banff Working group recommended to replace all these terms by “plasma cell-rich rejection” (PC-rich R) (3). We agree with the authors that AIH and PC-rich R are histologically very difficult to distinguish but fortunately, we have now a very specific serology pattern.

PC-rich R is a true rejection process that starts with the recognition of a donor antigen expressed in the graft by the recipient immune system. This is due to a genetic mismatch when the recipient lacks any copy of the Glutathione S-transferase T1 (GSTT1) gene and the donor carries at least one copy of this gene (4–6). Some of these mismatched patients develop a specific immune response by producing GSTT1 donor-specific antibodies, which is a required but not sufficient condition to develop PC-rich R. We have characterized anti-GSTT1 antibodies and the predominant IgG subclasses were IgG1 and IgG4 (7). Interestingly, IgG4 appear again involved in PC-rich R, this time as donor-specific antibodies.

It is clear that rAIH and PC-rich R represent distinctive clinical entities. The results presented in the article by Horwich et al. and the knowledge of the GSTT1 genetic mismatch with subsequent production of anti-GSTT1 antibodies (especially IgG4) should facilitate differential diagnoses between PC-rich R and other inflammatory post-transplant pathologies that have been particularly difficult when pre-LT disease was uncertain as mentioned by the authors.
